# The impact of psychological capital on nurses’ job performance: a chain mediation analysis of problem-focused coping and job engagement

**DOI:** 10.1186/s12912-024-01802-6

**Published:** 2024-03-02

**Authors:** Hao Chen, Nick Yvan Ngansom Kewou, Samuel Atingabili, Ary Dylann Zeudong Sogbo, Armel Temagna Tcheudjeu

**Affiliations:** 1 School of Management, Jiangsu University, Zhenjiang, Jiangsu China; 2 Department of General Surgery, School of Medicine, Jiangsu University, Zhenjiang, Jiangsu China

**Keywords:** Psychological capital, Problem-focus coping, Job engagement, Job performance, Chain mediation, Cameroon, Healthcare workers

## Abstract

**Background:**

Previous studies have explored the relationships of psychological capital with employees’ job performance in the health sector. However, the possible indirect pathways, including a serial mediation of problem-focus coping and job engagement, have not been extensively examined. This article explores how psychological capital influences nurses’ coping strategies focused on problem-solving, their level of engagement with their jobs, and how this, in turn, affects their job performance.

**Methods:**

The study involved 575 nurses from Cameroon’s public health sector. It investigated how psychological capital, an intrinsic resource, triggers nurses’ problem-focus coping liaison with job engagement to impact job performance. Analysis was conducted to assess the relationships among psychological capital, problem-focus coping, job engagement, and job performance with the use of SmartPLS 4.0 and PROCESS 4.2.

**Results:**

Findings revealed a significant effect of psychological capital on problem-focus coping, job engagement and job performance. Moreover, notable relationships were identified between psychological capital, problem-focus coping, job engagement, and performance, highlighting a chain mediation effect.

**Conclusion:**

The research advocates for hospital managers to employ strategies fostering employees’ psychological capital to better cope with organizational stressors to promote job engagement and enhance job performance. The study contributes fresh insights into healthcare organizational dynamics and human resource management, providing a foundation for future advancements in this field.

## Introduction

The well-being and job performance of healthcare workers are of utmost importance for healthcare organizations worldwide. Healthcare workers often face high demands and stress, which can have negative effects on their engagement, productivity, and the quality of care they provide if not properly managed [1]. Psychological capital (PC) is a valuable resource that assists nurses in effectively dealing with challenges and maintaining their level of engagement [2]. It comprises the psychological capacities of hope, resilience, optimism, and self-efficacy [2]. PC is the concept of self-identity and pertains to individuals’ confidence in their capability to overcome difficult situations. Optimistic individuals use their cognitive abilities to pursue goals and handle challenging circumstances. Those with hope are inclined to find alternative routes to achieve their objectives, while resilient individuals are characterized by their ability to bounce back from adversity and uncertainty [3]. Nurses who possess high levels of positive psychological capital exhibit greater engagement at work and demonstrate higher levels of job performance [4].

Understanding coping mechanisms is crucial in comprehending how individuals navigate stressful situations. Studies on coping mechanisms have identified two distinct types: problem-focused coping and emotion-focused coping. Problem-focused coping involves cognitive and behavioral strategies intended to modify stressful circumstances, including seeking information, brainstorming potential solutions, selecting the most suitable solution, and taking appropriate actions. Conversely, emotion-focused coping encompasses strategies aimed at managing the emotional responses elicited by stressful situations [5]. It is worth noting that previous research has consistently shown that problem-focused coping tends to yield more favorable outcomes in terms of well-being and performance compared to emotion-focused coping [6]. For this reason, the study adopted problem-focused coping to be a mediator in the nexus between PC and JP.

Furthermore, previous studies have explored the relationship between psychological capital, problem-focused coping, and various outcomes. For example, In a structural equation model analysis, E Rabenu, E Yaniv and D Elizur [6] found a significant mediation effect of coping through change between employee psychological capital and performance. F Saleem, MI Malik and SS Qureshi [7] investigated the connection between positive psychological capital and job stress, finding that problem-focus coping mediated this relationship among high school students in Pakistan. Similarly, problem-focus coping was found to mediate the relationship between psychological capital, family support, and student well-being in a study using cross-lagged mediation analysis [8]. Another study conducted in Hong Kong assessed the mediating roles of psychological capital and problem-focus coping between students’ social support and academic performance [9]. Also, a study conducted with 350 nurses across four public hospitals employed structural equation modeling and found that job embeddedness mediates the relationship between psychological capital and employee performance [2]. In a recent study among Chinese educational organizations employees, findings revealed that authentic leadership positively influences job performance (JP) directly and indirectly through PC, with perceived organizational support moderating the impact of authentic leadership on PC [10].

However, despite the existing literature, there is a research gap concerning the impact of psychological capital on problem-focused coping, job engagement, and job performance, specifically in the healthcare sector. Most studies have focused on the educational sector, overlooking the need for healthcare workers to cope with mounting stress while maintaining job engagement and preventing declines in performance. The emphasis has been disproportionately on boosting performance rather than strengthening coping strategies to enable continued work dedication under rising pressures.

Although job engagement and job performance are commonly conceptualized as outcome variables rather than mediators in most studies, this extant study proposed job engagement (JE) as a mediating variable in this study. Job engagement refers to a positive and fulfilling emotional and motivational state related to work well-being [11] linked to job performance [12]. The level of work engagement can change over time based on situational factors and the presence of personal resources such as PC [13]. In this research, insights from the conservation of resources (COR) theory [14] and the job demands-job resources (JD-R) model [15] are employed to construct and evaluate a dynamic motivational process model. This model aims to establish the relationship between PsyCap, work engagement, and, subsequently, job performance. Emerging research supports the notion that job engagement acts as an explanatory mechanism through which personal resources like psychological capital impact job performance [16, 17]. Studies have found psychological capital increases work engagement, which then leads to reduced intentions to quit and potentially improved performance [18]. While both engagement and performance are outcomes, engagement represents an intervening psychological state that occurs prior to performance.

This study aims to address this research gap by examining the relationships between psychological capital, problem-focused coping, job engagement, and job performance in the healthcare industry. Employing a chain mediation analysis, it aims to explore how problem-focused coping mechanisms impact job performance within the specific cultural framework of healthcare organizations. By delving deeply into these dynamics and employing a chain mediation framework, this research endeavors to offer valuable insights for healthcare leaders and administrators striving to bolster the well-being and productivity of their workforce amid organizational challenges and the demanding healthcare environment.

Lastly, this study seeks to expand the scope of knowledge in organizational psychology and healthcare management by offering a comprehensive comprehension of the interactions between these variables. The insights garnered from this research can offer actionable implications for healthcare managers and practitioners, presenting strategies to nurture employees’ psychological capital, manage organizational stressors, and foster increased job engagement and performance levels. By employing the Conservation of Resources Theory and Job Demands-Resources Model, this study contributes theoretically by investigating how psychological capital influences coping strategies, engagement, and overall outcomes, specifically within the healthcare sector.

## Literature review and hypothesis development

In this study, the combination of the JD-R (Job Demands-Resources) model and the COR (Conservation of Resources) theory provides a comprehensive theoretical framework for investigating the impact of psychological capital on nurses’ job performance, mediated by problem-focused coping and job engagement. The COR theory, proposed by SE Hobfoll [14], emphasizes the importance of resources and how individuals invest and protect them to effectively manage stressful situations.

Problem-focused coping, as conceptualized by RS Lazarus [19], represents a constructive approach to stress management, wherein individuals rely on their abilities to address and handle stress-inducing circumstances effectively. This research builds upon the COR theory by recognizing that problem-focused coping, along with four personal resources (self-efficacy, hope, optimism, and resilience), collectively forms psychological capital. These resources are considered as investments that individuals make to cope with stress and challenges [14]. The JD-R model incorporates these personal resources as positive factors within the broader framework of job demands and job resources. The study explores problem-focused coping as a psychological resource that nurses protect, preserve, and accumulate, leading to positive outcomes such as increased engagement and, subsequently lead to job performance [14, 20].

The JD-R model, proposed by Bakker and Demerouti, offers a positive perspective on stress by considering both the health-enhancing and health-impairing aspects of the workplace [21]. This healthcare setting presents job demands that could deplete nurses’ resources according to COR theory through the health impairment process. While previous stress models focused primarily on negative aspects like high workload and inadequate rewards, leading to negative consequences for individuals, the JD-R model goes beyond that. It not only examines the negative aspects but also considers the positive job characteristics and their influence on organizational outcomes through the motivational process.

The integration of the JD-R model and the COR theory is particularly relevant for this study. Firstly, both frameworks recognize the importance of resources as determinants of threats/demands and negative outcomes. The JD-R model identifies job resources that can buffer the negative impact of job demands, while the COR theory highlights the role of personal resources, including problem-focused coping, in resource accumulation [14, 21]. Secondly, the motivational process described in the JD-R model aligns with the resource accumulation assumption of the COR theory. The JD-R model explains how resources, including psychological capital, are accumulated and utilized to generate positive organizational outcomes, which is consistent with the resource conservation process proposed by the COR theory [14, 20].

Additionally, the JD-R Model shares common ground with the COR theory, providing a theoretical basis for our study. Both theories apply a demands-resources framework and contend these interact to influence important individual and organizational outcomes, as will be tested in this research. By combining the JD-R model and the COR theory, this study aims to provide a comprehensive understanding of the relationships between psychological capital, problem-focused coping, job engagement, and, ultimately, job performance. It recognizes the interplay of job demands, job resources, and personal resources in influencing nurses’ coping strategies, engagement levels, and overall job performance.

### Job demand resources model

The JD-R model was introduced in 2001 and proposes that all workplace characteristics can be classified into two categories - job demands and job resources [22]. Job demands refer to physical, psychological, social or organizational aspects of the job that require effort and are associated with physiological and psychological costs. Examples are high workload and emotionally demanding interactions. On the other hand, Job resources refer to aspects of the job that help achieve work goals, reduce demands, and stimulate growth and development. Examples are autonomy, feedback and development opportunities [23]. The JD-R model proposes two key processes - a health impairment process whereby high job demands lead to exhaustion and health issues, and a motivational process whereby job resources enhance motivation and engagement (Proposition 2) [22]. Job resources can buffer the negative impact of job demands on strains like burnout (Proposition 3), particularly when demands are high (Proposition 4) [24]. Personal resources like optimism and self-efficacy play a similar role to job resources in the motivational process (Proposition 5) and can buffer the stressor strain relationship [25]. Motivation has a positive effect on performance, while strain has a negative effect (Proposition 6) [11]. Engaged employees proactively optimize demands and resources through job crafting, leading to more resources and engagement (Proposition 7) [16].

The utilization of the job demands-resources (JD-R) model [23] can offer insights into the relationships among psychological capital, problem–focused coping, job engagement, and job performance. According to the JD-R model, there exists a motivational process wherein resources such as psychological capital and problem–focused coping contribute to engagement in work, which subsequently leads to improved employee performance and outcomes. Previous research has explored this mediating process in organizational contexts, demonstrating that work engagement mediates the impact of PC on organizational citizenship behavior [26]. By applying the JD-R model to health settings, it is reasonable to expect that psychological capital would lead to a direct effect on job performance while problem–focused coping and job engagement, which, in turn, would have a mediation effect on the relationship between PC and PFC outcomes.

### Conservation resource theory

The Conservation of Resources (COR) theory [14] provides a useful lens for examining the impact of organizational politics on health workers’ job engagement and performance [27]. COR posits that individuals strive to obtain, retain, protect, and foster valued resources. Resources are defined broadly as objects, conditions, personal characteristics, and energies that people draw upon to function effectively [14]. When resources are threatened or depleted, individuals experience stress and adopt a defensive posture to conserve remaining resources. When resources are threatened or depleted, individuals experience stress and adopt a defensive posture to conserve remaining resources. According to COR, individuals need to acquire and conserve resources regardless of the obstacle they face, be it stressful or not. They make use of their key personal resources to obtain new resources further. The repetition of this mechanism within an individual creates a resource caravan [28]. Henceforth, they make use of resources such as resilience, self-efficacy, optimism, and hope to cope, overcome obstacles and maintain new resources. This gives individuals the sense of facing and withstanding all stresses and difficulties encountered. For instance, an employee with a high psychological capital can easily cope with a problem by perceiving it as an opportunity or an obstacle that he or she must challenge to obtain resources.

In the workplace, COR theory suggests that organizational stressors can threaten employees’ resources like status, autonomy, belongingness, and self-esteem [29] perceived as problems by employees. Hence, they tend to put in place defensive measures, which are problem-focused coping strategies. Problem-focus coping strategies are not only considered as stress shields but equally considered as a source of energy capable of triggering job engagement [30] and job performance [31]. Psychological capital can consequently alter individual problem-focused coping, consequently affecting traditional work outcomes. This phenomenon shall be most effective in a supportive environment where organizational stakeholders strive in one way or the other to affect employees’ psychological capital [32].

Conversely, positive psychological resources can counteract resource depletion. For example, psychological capital characterized by hope, self-efficacy, resilience, and optimism can act as a reservoir of strengths, enabling employees to thrive despite adversity [33]. Workers with greater PC are less likely to perceive their work context as threatening or unjust, mitigating a sense of stress. PC also equips people to cope with challenges proactively. This suggests PC may increase problem-focus coping and buffer against associated declines in engagement and performance predicted by COR theory [34].

### The nexus between psychological capital and problem-focus coping

Humans exhibit adaptability and harness their resources to navigate challenging circumstances by drawing on their psychological capital. When faced with difficult or adverse situations, individuals can, at a minimum, endure the impact of such situations, provided they possess the necessary means to combat them [35]. Organizational stressors are often considered antecedents to employees’ inappropriate attitudes and behaviors within the organization [36, 37]. Also, workers with a positive psychological capital tend to see challenges as growth opportunities due to their strong problem-focused coping skills. This mindset stems from their adeptness in handling stress and difficult situations [38]. Employees frequently use their resources to engage in problem-focused coping mechanisms in response to organizational pressures to preserve their equilibrium [38]. These resources include resilience, which enhances their energy-restoring and accelerating abilities [39, 40], enabling them to gain strength, return to a state of well-being and rebound from adverse situations [41], thereby fostering positive outcomes and advancing their future progress [42]. F Liang and L Cao [43] confirmed in their study that the relationship between resilience and problem-focused coping is significant.

Furthermore, individuals with robust self-efficacy are more likely to possess the confidence to direct their efforts toward achieving their goals. A Bandura, WH Freeman and R Lightsey [44] highlighted that individuals who have confidence in their abilities to manage situations are more inclined to meet challenges head-on rather than walk away from them or feel overpowered by them. Additionally, a positive correlation was found between self-efficacy and problem-focused coping [45]. Optimism has been identified to play a significant role in shaping a person’s positive affective evaluation. Employees’ problem-focused coping style is fueled by optimism, which enables them to approach obstacles with positive energy and actively seek out possibilities for development [46]. Optimistic individuals are more inclined to persevere and persist in the face of challenging circumstances because they anticipate positive outcomes, remaining steadfast despite obstacles [47, 48]. Optimism acts as a resilience-enhancing factor when confronted with daunting situations, promoting goal-directed behavior among workers [48]. In his study, D Strutton and JR Lumpkin [49] discussed the weightiness of optimism in coping strategies. Following AK Yahsa, I Rahayuningsih and N Laily [50] results on the positive relationship between optimism and problem-focus coping.

Moreover, individuals who demonstrate personal resources, such as hope, possess the ability to adapt and leverage their various resources to meet the demands of stressful situations [20] effectively. Hope leads individuals toward problem-focused coping by encouraging belief in possibilities and a strong will to overcome obstacles with grit and perseverance [51]. An empirical study by EC Chang [52] found a correlation between hope and problem-focused coping. Numerous studies amalgamate these elements into psychological capital, examining how this composite measure influences problem-focused coping strategies, showcasing how an individual’s hope, optimism, resilience, and self-efficacy collectively shape their approach towards addressing challenges with proactive problem-solving techniques [8, 53–55], the association between the two concepts is an open secret.

Considering this literature, it would be intriguing to explore the influence of psychological capital on problem-focus coping. As a result, we propose the following hypothesis:

#### Hypothesis 1

*Psychological capital positively influences nurses’ problem-focused coping.*

### Relationship between psychological capital and job engagement

Employee engagement represents the extent to which workers invest their full selves—physically, emotionally, and cognitively—into their roles [56]. It can be seen in the thoughts, feelings, and behaviors of employees as well as in how emotionally connected they are to their organization [57]. Engaged employees exhibit high energy, involvement, dedication, and absorption in their work. Within healthcare settings, nurse engagement has been linked to better patient outcomes, fewer errors, and higher care quality [58]. Researchers have increasingly focused on positive organizational resources, like psychological capital (PC), as antecedents that can enhance employee engagement across occupations. The interpretation of the concept of job engagement refers to how dedicated a worker is to assisting their company in achieving its objectives [59]. Employees who manifest positive psychological capital and positively interpret tasks easily experience a positive emotional state, which broadens their acquainted behavioral patterns and encourages their engagement in personal tasks [60]. Psychological capital may give workers a more positive outlook on setbacks, high energy, and a resilient mentality to overcome obstacles as a result of their vigor, which favors their level of job engagement [61].

PC encompasses one’s hope, optimism, resilience, and self-efficacy [33]. Multiple studies have proposed and demonstrated a positive relationship between PC and job engagement. For instance, a study of Chinese teachers found that PC predicted engagement through its positive impact on work meaningfulness [62]. Teachers with greater PC found more meaning in their roles, which strengthened their engagement. The hope and self-efficacy facets of PC specifically increased willingness to invest effort. Employees with self-efficacy are considered to have a high sense of reasonability, excitement, and passion for their job, surrendering them and increasing their engagement [63]. D Sweetman and F Luthans [64] found that the combination of all four psychological components, self-efficacy, hope, resilience, and optimism, generates a positive spiritual element that enforces employee job engagement. A meta-analysis has shown that personal resources strongly influence job engagement [65]. Therefore, based on the aforementioned literature, employee psychological capital positively predicts their level of job engagement.

#### Hypothesis 2

*Psychological capital is positively related to nurses’ job engagement.*

### The association between psychological capital and job performance

Job performance is generally defined as employees’ effectiveness in carrying out work responsibilities that contribute to organizational goals and operations [66]. Job performance represents the overall quality and effectiveness of employees’ work contributions. Within healthcare, nurse performance impacts critical outcomes like patient satisfaction, mortality rates, and adverse events [67]. As such, understanding the drivers of healthcare worker performance has been a focal interest. Psychological capital (PC) has emerged as an important individual resource that may enhance employees’ performance. It was found by F Luthans, BJ Avolio, FO Walumbwa and W Li [68] in a study on Chinese employees that hope, optimism, and tenacity as a whole in psychological capital act as an antecedent to predict job performance positively. In a study on the effect of psychological capital on the job performance of employees in Public institutions, privately owned businesses, and state-owned businesses in the Qingdao region of Shandong Province, China [4]. The study found that psychological capital, through its components of self-efficacy, optimism, hope, and resilience, positively impacts job performance. These personal resources act as the perfect antecedent because individuals usually make use of their motivation stimulations, which is favored by their psychological capital, to enhance their creative and innovative behavior [69] since they manifest self-efficacy, which causes them to be prompt in adapting themselves, powerfully, productive, and accurate in preventing organizational resources wastage [70].

On the other hand, optimism gives employees the capacity to fulfill important duties and obligations, often fostering individual job performance [71]. Homogenously, individuals with high levels of hope frequently adopt agency thinking, having affirmations like “I can handle this”, which intends to foster their resilient behavior to take action to solve problems and increase performance [72]. Hope is barely considered a task performance instigator [73]. As a task performance instigator, researchers found that employees with a high psychological capital tend to perform better regardless of their perceived level of job stress [74]. A growing body of empirical evidence indicates PC can act as a significant predictor of work performance across cultural contexts [75]. Evaluating this relationship among healthcare professionals in Cameroon would meaningfully extend this research. However, it is hypothetical to infer from the clarification mentioned above that individuals who express positive psychological capital will exhibit high job engagement and performance. Thus, the following hypotheses are put forth in this study:

#### Hypothesis 3

*Psychological capital is positively related to nurses’ job performance.*

### The nexus between problem focus coping and job performance

When individuals actively employ problem-focused coping (PFC) strategies in their approach to work, it tends to yield a noticeable improvement in their performance [31]. This occurs because they efficiently confront and handle challenges as they arise, fostering a more productive work environment [76]. Their adeptness in addressing these challenges not only boosts their efficiency but also contributes to the successful resolution of tasks, ultimately enhancing their overall performance [76] within their professional roles. Problem-focus coping techniques are viewed as auxiliary evaluations that represent proclivities toward action, to alter the individual interactions with the environment throughout the coping process. There’s a continuous stream of emotions—stemming from the demands posed by the task at hand [77]. In an empirical study, GD Sideridis [77] discussed that individuals use their problem-focus coping strategy to trigger their emotions to achieve and meet their task demands.

Similarly, in a meta-analysis study carried out in 3 major cities of China, L Lu, SF Kao, OL Siu and CQ Lu [31] found that coping strategies are related to employee job performance. Existing literature has demonstrated a connection between coping strategies and psychological well-being as well as overall functioning [78, 79]. Since individuals often choose a specific coping approach based on the context and the particular challenges they are facing, often employees who favourably perceive and cope with organizational stressors foster their personal development, motivation [80], and overall performance. Therefore, from the literature mentioned above, individuals who engage in problem-focused behaviors perceive stressful situations as an opportunity to manifest high job performance. Thus, the following hypothesis is put forth in this study:

#### Hypothesis 4

*Problem-focused coping is positively related to nurses’ job performance*.

### The relationship between job engagement and job performance

According to WB Schaufeli, AB Bakker and M Salanova [81], job engagement is a sustained, favorable, affective, motivational state of fulfillment characterized by vigor, dedication, and absorption. This explains the reason why engaged employees are enthusiastic about their work and have high levels of energy [82]. Employees who are highly engaged in their job tasks, on the whole, put a lot of physical effort into achieving role-related objectives while also being alert cognitively and emotionally. SP Brown and TW Leigh [83] found in several samples a relationship between workers putting in more effort and better job performance. Similarly, BL Rich, JA Lepine and ER Crawford [84] stated that increasing employee engagement is likely to encourage workers, which will lead to better job performance. Empirically, G Yongxing, D Hongfei, X Baoguo and M Lei [85] indicated that work engagement is positively related to objective task performance. We have compelling theoretical justifications for supposing that there is a relationship between job engagement and job performance. Therefore, based on the above literature, the following hypothesis was put forth.

#### Hypothesis 5

*Job engagement is positively related to nurses’ job performance*.

### The mediating role of problem-focus coping on the relationship between psychological capital and job performance

Psychological capital has demonstrated positive associations with employee performance across cultural contexts [33]. The utilization of personal coping resources and strategies to improve performance is a path used by individuals to address both internal (negative emotions) and external (failures) pressures during stressful situations [86]. Individuals possess the ability to navigate challenging situations by effectively managing stress, meeting demands, problem-solving, and utilizing positive thinking and emotional expression to handle stressful circumstances [87]. Problem-focused coping was empirically found to be a mediator between psychological capital and wellbeing [8]. Similarly, OL Siu, BCY Lo, TK Ng and H Wang [53] in their study assessed the mediating role of problem focus coping between students’ psychological capital and their outcomes, including academic performance. However, room is still left by literature to research the mediating role of problem-focused coping and its capability of triggering favorable organizational outcomes within different sample bodies.

According to the Conservation of Resources theory, PC represents a key resource reservoir that may reduce perceptions of organizational stressful circumstances as threatening or unjust [88]. Employees who exhibit high PC tend to perceive their work environments more positively and feel equipped to navigate challenges. In turn, this lowers the negative ideology of stress and instead preserves motivation and effort directed toward job performance. PC enables employees to channel their energies into work-related activities. PC resources like optimism and resilience reduce individual perceptions of stress adversities, but rather an energy self-serving behavior, preserving employee performance. Therefore, it is ideal to hypothesize that employees who exhibit positive psychological capital advantageously perceive and cope with workplace challenges and improve their job performance. For this reason, the study hypothesizes that.

#### Hypothesis 6

*Problem-focused coping will mediate the impact of psychological capital on job performance.*

### The mediating role of job engagement between psychological capital and job performance

Job engagement is conceived as the amount of time, energy, and commitment a person puts into their work is an expression of their level of motivation at work [59]. Job performance, on the other hand, is a behavioral outcome that the organization recognizes and values [84]. Therefore, personal potential (i.e., psychological capital and positive changes in psychological capital) are often considered great instigators of productive organizational behaviors that effectively improve job performance through the intrinsic motivational process of improved work engagement [89]. G Alessandri, C Consiglio, F Luthans and L Borgogni [90] reported that the idea of job engagement being an antecedent of job performance acknowledged that job engagement can act as a mediator between psychological capital and job performance. Similarly, C-Y Chen [91] suggested conceptualizing job engagement more as a mediator between psychological capital and job performance.

In addition, emerging research supports job engagement as an explanatory mechanism in the PC and job performance nexus. For instance, OS Olaniyan and SW Hystad [92] revealed a direct relationship between authentic leadership and followers’ job satisfaction, psychological capital, and work engagement and an indirect relationship between authentic leadership and intention to quit through employee psychological capital and work engagement. The study found psychological capital increased work engagement, which in turn reduced intentions to quit, supporting the mediating role of engagement. It did not directly test effects on task performance, as mentioned in the example, but provides related evidence for the mediated pathway through engagement. PC is, therefore, expected to boost employees’ willingness to devote their full selves to delivering high-quality care. Similarly, research on frontline retail employees showed that team PC elevated service performance through increased work engagement [34]. Shared PC resources within teams strengthened members’ involvement and enthusiasm, driving higher customer satisfaction ratings.

High job engagement could be attained with a positive psychological capital mindset to boost greater levels of employee performance in the current challenging workplaces. In light of the previous justification, the following hypothesis is put forth:

#### Hypothesis 7

*Job engagement will mediate the impact of psychological capital and job performance*.

### The chain mediation effect of problem-focus coping and job engagement on the nexus between psychological capital and job performance

Following the Conservation of Resources (COR) theory, individuals possess internal reserves that drive them to safeguard their existing resources and seek new ones, even in the face of stressful conditions [27]. The stressful scenario in question here is workplace challenges, which individuals perceive as subjective and a matter of mindset [93]. Individuals with a problem-coping nature can be readily influenced by the repeated display of positive psychological traits such as self-efficacy, optimism, hope, and resilience [94]. These traits instill motivational tendencies that can significantly impact individual opportunistic perceptions of difficulties. On one hand, individuals may see it as a challenge to overcome [95], while conversely, they may choose to disregard the situation entirely. In both cases, this heightened engagement. Behavioral and cognitive approaches to coping were empirically found as predictors of engagement [30]. In the pursuit of overcoming challenges, employees tend to foster positive emotions (e.g., enthusiasm, excitement, and ecstasy) and adopt a proactive, problem-solving coping attitude (e.g., planning and exerting extra effort) [95]. DR May, RL Gilson and LM Harter [96] propose that the substantial manifestation of positive emotions and the experience of deriving meaning from challenges are strongly associated with high levels of engagement, which have been empirically proven to be predictors of job performance [84, 97].

Problem-focused coping and job engagement act as important mediators in the relationships between psychological capital and job performance. Drawing on the theoretical frameworks of the Job Demands-Resources (JD-R) [21] model and Conservation of Resources (COR) [14] theory. The JD-R model proposes that job resources (personal resources) like psychological capital lead to motivational processes and higher job engagement. COR theory suggests that resources can start gaining spirals, where resources accumulate and generate further resources. When applied to nurses, psychological capital acts as a key personal resource that can initiate upward gain cycles toward greater problem-focused coping abilities and job engagement.

Research evidence indicates that psychological capital boosts problem-focused coping, an active coping strategy focused on resolving issues [8]. Problem-focused coping then acts as a mediator to improve nurses’ outcomes like job performance, well-being and behavioral conduct. This is because the self-efficacy, hope, resilience and optimism associated with psychological capital enable nurses to gain problem-focused coping resources to handle work-related stressors proactively.

Furthermore, studies show that psychological capital increases job engagement, which in turn improves job performance [9]. This suggests the resources help create motivational pathways leading to higher job engagement, which subsequently mediates effects on job performance. In summary, the JD-R model and COR theory propose that resource caravans and gain spirals can occur, whereby resources like psychological capital accumulate and generate further resources like problem-focused coping and job engagement. These new resources then act as mediators to positively impact nurses’ well-being, job performance, and behavior. To test the sequential chain mediation effect of problem-focused coping and job engagement on the nexus between psychological capital and job performance, this study suggests the following:

#### Hypothesis 8

*Problem-focused coping and job engagement play a chain mediating role between psychological capital and job performance*.

**Fig. 1:**
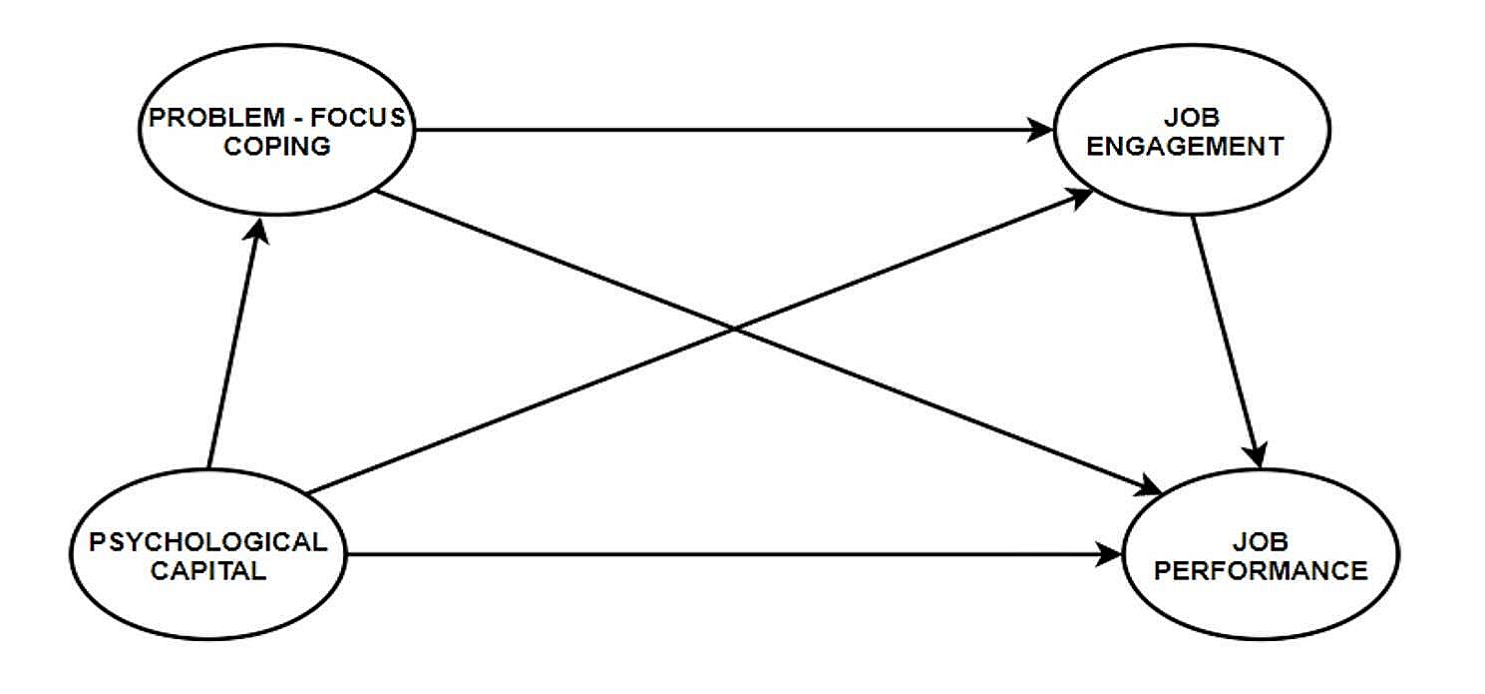
Conceptual framework

### Research model

The research model underlines the relationship between psychological capital (manifested as self-efficacy, hope, optimism, and resilience), perceived organizational politics, job engagement, and job performance, as shown in Fig. 1. As displayed by the above model, psychological capital influences employees’ problem-focused coping, job engagement, and job performance. Problem-focused coping and job engagement form a chain mediation that influences the relationship between psychological capital and job performance. Age and work experience are used as control variables to investigate if the latter can influence the significance of the chain mediating effects between our studied variables.

## Material and method

### Sample and procedure

The study sample focused on only two of the central hospitals in Cameroon. These two are considered to be the two largest hospitals in the country with a relatively high amount of nurses. Selecting these two central hospitals in Cameroon allows for an in-depth exploration of how psychological capital influences nurses’ job performance within the country’s diverse healthcare landscape. Studying nurses in these settings enables a comparative analysis that reveals insights into problem-focused coping and job engagement, enriching our understanding of factors impacting nurse performance in Cameroon.

A cross-sample was used as a way to manage the biases in the responses. The health institutions were contacted with an official letter. Questionnaires were distributed to nurses and their direct supervisors in sealed envelopes. Nurses and their direct supervisors sealed the responded questionnaires and placed them into boxes at their workplace assigned for the survey. This procedure was used to ease the data matching realized through code identification. To match the nurse and supervisor questionnaires while preserving confidentiality, we implemented an identification code system. The human resources department of the two hospitals provided the researcher with lists of all the nurses, with each nurse assigned a unique identification code. The same identification codes were marked on the questionnaires distributed to each corresponding nurse. Supervisors were assigned identification codes as well, which were marked on their questionnaires. By including matched identification codes on both the nurse and supervisor questionnaires, we were able to pair each employee’s responses with their supervisor’s responses for data analysis. This coding system enabled the pairing of questionnaires to compare responses while keeping the participants’ identities strictly confidential throughout the process. A total of 575 out of 660 nurses to whom the questionnaire was initially assigned fully completed the questionnaire with items that intended to measure psychological capital, problem-focus coping and job engagement, while 49 direct supervisors completed questionnaires about each of the nurses’ job performance. For a total response rate of 87%, this data collection procedure is consistent with [98, 99]. Table 1 gives details of the descriptive analysis of respondents.

**Table 1:** Descriptive analysis of respondents

Respondent		(*n* = 575) profile	
Attributes	Category	Frequency	Percentage
Gender	Male	213	37%
	Female	362	63%
Age	18–27	182	32%
	28–37	227	39%
	38–47	98	17%
	48–57	47	8%
	58 and above	21	4%
Education Level	Secondary and below	55	10%
	Diploma	134	23%
	Bachelor	267	46%
	Masters and above	119	21%
Level of experience	Less than 1 year	62	11%
	1–5 years	176	31%
	6–10 years	197	34%
	11–15 years	88	15%
	Above 15 years	52	9%

Based on studies that were published and considered significant to our investigation, the questionnaire was created. It was divided into two sections, the first of which examines the respondents’ general characteristics (such as gender, age, education, and years of experience), and the second of which specifically looks into the study variables, which are psychological capital (PC), Problem-Focus Coping (PFC), job engagement (JE) and job performance (JP)

### Variable measurement

Psychological capital was developed by F Luthans, CM Youssef and BJ Avolio [33] with 24 items, and the latter was adopted for the sake of this research. Of the 24 items, Hope, Self-efficacy, Resilience, and Optimism each have 6 items with a 5-point Likert scale ranging from (1) ―strongly disagree to (5) ―strongly agree was used to evaluate the replies with consistency. Sample items included “At present, I am energetically pursuing my work goals;” “I feel confident helping to set targets/goals in my work area;” “When I have a setback at work, I have trouble recovering from it and moving on;” “When things are uncertain for me at work, I usually expect the best”.

Problem-focus Coping was evaluated using the 9 items of RSF Lazarus, S [100]., which involved actions (cognitive and behavioral) intending to improve or resolve stress-eliciting situations. A 5-point Likert scale ranging from (1) ―strongly disagree to (5) ―strongly agree was used to evaluate the respondent’s replies with consistency. Some of the items measured included “I come up with several alternative solutions to the problem” and “I make a plan and follow it”.

Job engagement was measured with a 7-item scale adapted from M Obschonka, I Pavez, T Kautonen, E Kibler, K Salmela-Aro and J Wincent [101] a five-point Likert scale ranging from (1) strongly disagree to (5) strongly agree was used to assess each item of this variable. Some of the items included “When I work, I feel full of energy;” and “I am enthusiastic about my work”.

The job performance was measured using the 5 items adopted from [102] confirmed and used in recent studies [98]. All the constructs were measured on a 5-point Likert scale to grade the responses. Some of the items were “He/she is recognized as one of the best employees” and “He/she selected among the top 10% of employees in the workplace.”

### Data analysis

The research followed a structured three-step data analysis approach. Initially, descriptive and correlation analyses were executed using SPSS 23.0 to examine the sample features and correlations between the variables. Subsequently, Smart-PLS 4.0 was employed to perform confirmatory factor analysis and path analysis to assess the hypotheses. Finally, PROCESS version 4.2 was utilized to investigate simple mediation and chain mediation effects. The study used employee’s age and level of experience as control variables as it was discussed in previous studies that they have significant effects on subordinate performance [103]. G Yongxing, D Hongfei, X Baoguo and M Lei [85] stressed that age and experience have had a significant impact on employee performance, therefore, this study incorporates experience and age as control constructs of the study.

### Common method bias (CMB)

When data is gathered using self-report scales, a common method error may happen. Our study assessed the level of bias using the CMB method, the reason being that the current study model has to do with constructs related to respondents’ psychology, which can easily vary depending on the respondents’ mental state. According to this method, for the data to be free of common method error, the variance inflation factor (VIF) should be greater than 3.3 [104]. After analysis, our model was considered to be free of common method error since the VIF in the inner model resulting from full collinearity of all the items ranged between (1.369 and 2.983) see Table 2.

**Table 2:** Scale items and measurement model results

Constructs	Items	Loadings	CA	CR	AVE	VIF
Psychological capital	PC-SE1	0.678	0.953	0.955	0.585	1.980
	PC-SE2	0.712				2.066
	PC-SE3	0.662				1.410
	PC-SE4	0.783				1.725
	PC-SE5	0.803				2.350
	PC-SE6	0.782				2.161
	PC-HO7	0.889				1.979
	PC-HO8	0.885				2.801
	PC-HO9	0.740				1.816
	PC-HO10	0.850				2.496
	PC-HO11	0.858				2.560
	PC-HO12	0.674				1.489
	PC-RES13	0.747				1.930
	PC-RES14	0.809				1.502
	PC-RES15	0.805				2.312
	PC-RES16	0.761				2.152
	PC-RES17	0.695				1.997
	PC-RES18	0.751				1.780
	PC-OPT19	0.812				2.000
	PC-OPT20	0.825				2.134
	PC-OPT21	0.688				1.563
	PC-OPT22	0.750				1.923
	PC-OPT23	0.750				1.924
	PC-OPT24	0.780				1.897
Problem-focus coping	PFC1	0.700	0.877	0.885	0.510	1.684
	PFC2	0.834				2.447
	PFC3	0.706				2.690
	PFC4	0.728				2.820
	PFC5	0.821				2.533
	PFC6	0.714				1.894
	PCF7	0.589				1.378
	PCF8	0.624				1.406
	PCF9	0.672				1.549
Job engagement	JE1	0.868	0.877	0.879	0.732	2.424
	JE2	0.889				2.675
	JE3	0.861				2.314
	JE4	0.802				1.753
Job performance	JP1	0.687	0.773	0.775	0.525	1.343
	JP2	0.687				1.357
	JP3	0.742				1.502
	JP4	0.756				1.541
	JP5	0.747				1.473

### Reliability and validity of the scale

The assessment of the model’s reliability and validity was conducted using PLS-Sem measurement analysis from Smart PLS 4.0 as recommended by [105]. Before evaluating the measurement model, the loadings and significance of the indicators were assessed [106]. The outside weight of the index should be more than 0.7, according to [107]. According to GF Khan, M Sarstedt, W-L Shiau, JF Hair, CM Ringle and MP Fritze [108], indicators with external loadings between 0.4 and 0.7 should only be considered for elimination if doing so improves the result. Three items from job engagement did not meet the threshold and, therefore, were deleted to be able to perform further analysis. These 3 items were deleted because they affected the internal consistency of the data.

Using Cronbach’s alpha, composite reliability (CR), Rho-a, and the commonality of each concept, the reliability of the mirrored assessment model was evaluated. M Tenenhaus, VE Vinzi, Y-M Chatelin and C Lauro [109] stated that alpha values larger than 0.7 are adequate for Cronbach’s alpha. Table 2 shows that the Cronbach’s alpha (**α)** for each variable was greater than 0.7, acknowledging the internal consistency of our data, due to Cronbach’s alpha (**α)** constraints, composite reliability (CR) whose threshold is considered to be greater than 0.7. According to RA Peterson and Y Kim [110] was measured to substantiate the reliability of the study. Table 2 shows that the composite reliability of our data was assessed and significant.

The average variance extracted (AVE) quantifies the variance explained by indicators relative to the total variance, including the measurement error. A minimum AVE value of 0.5 is required to signify that the construct accounts for at least 50% of the variance among its indicators [111]. After analysis, the AVE was attained with significant figures ranging between (0.510–0.732).

Discriminant validity was used to evaluate the construct validity. For this matter, we made use of the Fornell-Larcker criterion [112] and the heterotrait-monotrait correlation (HTMT) [113]. Based on the Fornell-Larcker criterion method, discriminant validity is obtained by assessing the square root of the average variance extracted construct’s coefficient. For this assumption to be achieved the on-diagonal coefficients should be greater than the off-diagonal. After analysis, the results indicated a satisfactory level of discriminant validity, as shown in Table 3. The heterotrait-monotrait (HTMT) method put forward the ratio of heterotrait-monotrait correlation. To obtain empirical evidence, the discriminant validity requires the heterotrait-monotrait (HTMT) coefficient to be less than 0.90. After evaluation, results revealed an adequate proportion of satisfaction see values in brackets in Table 3.

**Table 3:** Discriminant Validity (Fornell-Larcker criterion) and (Heterotrait-Monotrait) (HTMT)

	JE	JP	PFC	PC
JE	**0.855**			
JP	0.715 (0.825)	**0.724**		
PFC	0.711 (0.740)	0.703 (0.628)	**0.714**	
PC	0.610 (0.832)	0.523 (0.830)	0.701 (0.780)	**0.764**

Note: Psychological capital (PC), Problem-Focus Coping (PFC), Job engagement (JE), and Job performance (JP)

### Correlation between variables

As shown in Table 4, the mean and standard deviation coefficients of psychological capital, problem-focused coping, job engagement, and job performance added to that of the study control variables experience and age. Table 4 equally represents the correlation that exists between the studied constructs of our model; therefore, evidence can prove that there exist positive correlations between our constructs.

**Table 4:** Correlation matrix

Variable	MEAN	SD	AGE	EXP	PC	PFC	JE	JP	
AGE	2.816	0.8878	1						
EXP	3.245	0.9613	0.496^**^	1					
PC	3.0500	0.71445	0.108^**^	0.066	1				
PFC	3.1586	0.69906	0.101^*^	0.040	0.905^**^	1			
JE	3.2465	0.87726	0.125^**^	0.054	0.877^**^	0.869^**^	1		
JP	3.1506	0.75827	0.079	0.048	0.903^**^	0.806^**^	0.812^**^	1	

**. Correlation is significant at the 0.01 level (2-tailed)

*. Correlation is significant at the 0.05 level (2-tailed)

Note: Psychological capital (PC), Problem-Focus Coping (PFC), Job engagement (JE), Job performance (JP), and Experience level (EXP)

### Evaluation of the structural equation

In the structural equation analysis, we examined the relationship between constructs and the strength and quality of the model to validate our studied hypothesis. The structural equation model (SEM) [114] from SMART-PLS 4.0 was used to evaluate the direct relationships between our model constructs to find if they are statistically significant or not based on the path coefficients (β) values, t-statistics (*T*) and *p*-values (*p*) [115], as well as their coefficient of determination R-square (*R*^*2*^), size effects (*f*^*2*^) and predictive relevance (*Q*^*2*^). The coefficient of determination R-square (R^2^) measures the proportion of variance between the explained and latent variables. According to [116] R-square has to be greater than 0.10 coefficient considered as the threshold. Table 5 shows that JE, JP and PFC scored 0.800, 0.825 and 0.827, respectively. This study equally reported the f-square which is the effect size. According to [117], the thresholds for the interpretation of size effects are (f2 < 0.02 = small, f^2^ < 0.15 = medium, f^2^ < 0.35 = large). Table 5 shows that the inter-relationships between PC, PCF, JE, and JP effects size were large to the exception of the relationship effects of PC on JE, which registered a medium size effect PFC on JP, and JE on JP registered to have a small size effect. The model predictive relevance (Q^2^), which, according to J Henseler, CM Ringle and M Sarstedt [113] the value of Q^2^ should be greater than zero as a sign that external structure has predictive relevance to the endogenous structure under examination Table 5 shows via the constructs JE, JP and PFC model was predictively useful supported by the respective coefficients 0.767, 0.820 and 0.826.

**Table 5:** R-Square, F-square and Q-Square

Constructs	R2	R^2^ Adjusted	Q2	path	f2
JE	0.800	0.799	0.767	PFC → JP	0.014
JP	0.825	0.824	0.820	PC →JE	0.204
PFC	0.827	0.827	0.826	PC →PFC	4.783
				PC →JP	0.699
				JE → JP	0.016

Note: Psychological capital (PC), Problem-Focus Coping (PFC), Job Engagement (JE), and Job Performance (JP)

To assess the relationship between the studied constructs the path coefficient test was run. For this test, the bootstrapping technique was used as recommended by GTM Hult, JF Hair Jr, D Proksch, M Sarstedt, A Pinkwart and CM Ringle [111]. The confidence interval was set at 95%, while the sample size was set at 5,000. This technique contains numerical values (β) values, standard errors t-statistics (*T*), and *p*-values (*p*). The findings of the hypothesized relationships support that H1 PC has a strong positive relationship with PCF (*β =* 0.909, T = 133.466, *p* < 0.000). Per Table 6, there is equally empirical support for H2 depicting a strong positive relationship between PC and JE (β = 0.485, T = 11.720, *p* < 0.000) and a strong positive relationship between PC and JP represented by the path coefficient values (β = 0.922, *T* = 21.795, *p* < 0.000) supporting H3.

**Table 6:** Test of the relationship in the structural model

	Relationship	β	Standard error	T statistics	*P* values	Decision
H1	PC→ PFC	0.909	0.007	133.466	0.000	Supported
H2	PC →JE	0.485	0.041	11.720	0.000	Supported
H3	PC→ JP	0.922	0.042	21.795	0.000	Supported
H4	PFC→ JP	0.129	0.042	3.060	0.002	Supported
H5	JE →JP	0.117	0.040	2.932	0.004	supported

Note: Psychological capital (PC), Problem-Focus Coping (PFC), Job engagement (JE), and Job performance (JP)

**Fig. 2:**
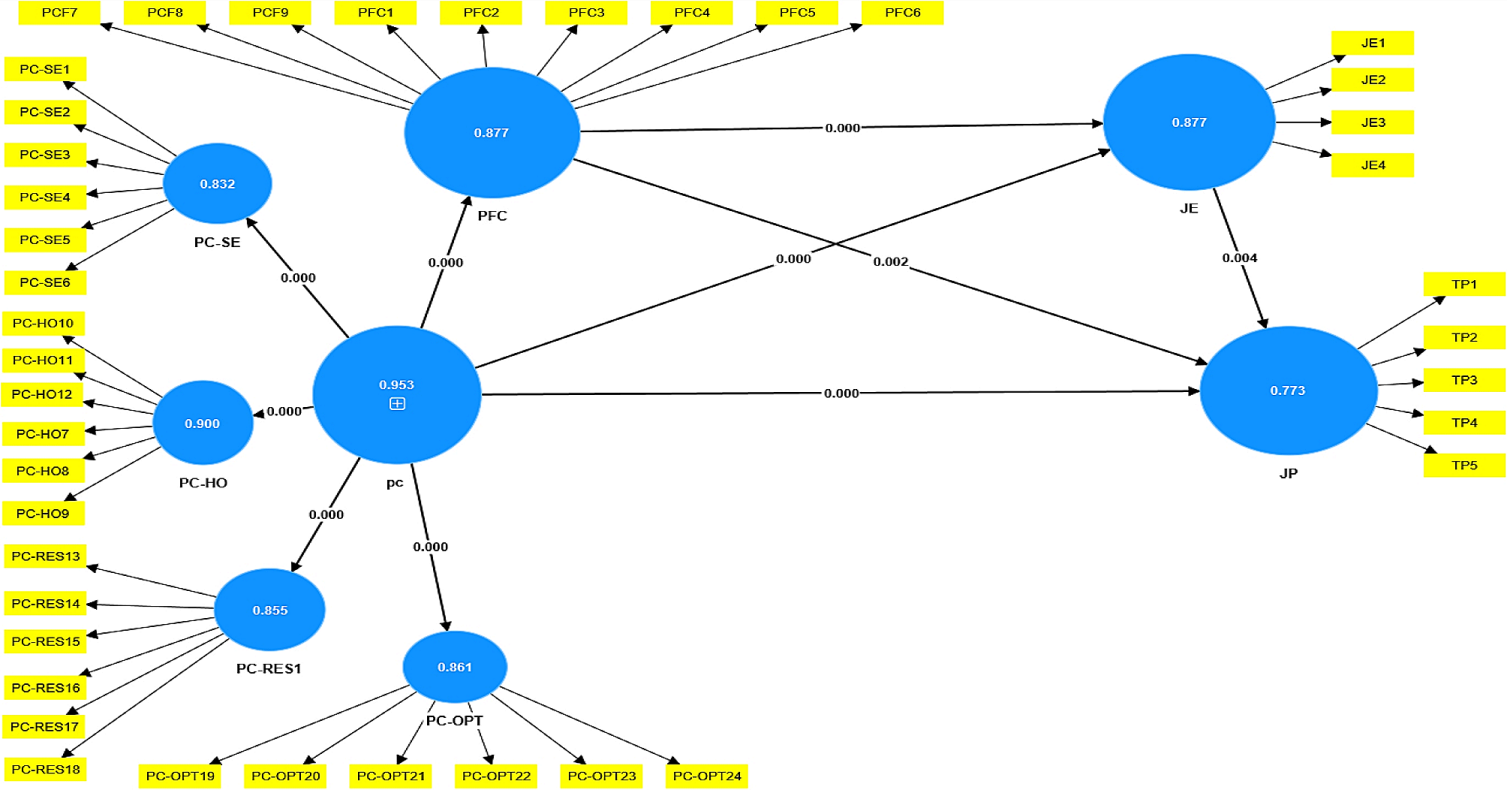
Results of the structural equation model

PCF is significantly and positively associated with JP (β = 0.129, T = 3.060, *p* < 0.002), hence empirically supporting H4. Again, there is a positive significant relationship between JE and JP (β = 0.117, t = 2.932 *p* < 0.004) hence supporting H5. The findings indicate that all pathways are statistically significant at a 5% significance level, as evidenced by *p*-values below 0.05 for all connections, affirming the validity of all hypotheses. After controlling for the participants’ ages and levels of experience, the findings revealed that nurses’ psychological capital has a significant impact on their performance. Refer to Fig. 2 for the graphical output of the SEM for the hypothesis testing in the study.

### Simple chain mediation analysis

This research adopted PROCESS version 4.2 to test the mediation effect of problem-focus coping and job engagement on the association between psychological capital job performances. Using 5,000 Bootstrap samples to help in the analysis. All confidence intervals were set to a 95% level of assurance using the Bias Corrected for Bootstrap CI technique. KJ Preacher and AF Hayes [118] proposed that if the interval between the lower coefficient of the confidence interval BootLLCI and the upper coefficient of the confidence interval BootULCI does not include 0, a statistically significant mediating effect is ascertained. Completing the analysis, the results demonstrated that the indirect effect of PCF between PC and JP was 0.0225. The confidence interval did not include 0 (Boot LLCI = 0.0037, Boot ULCI = 0.0268), indicating that the mediating effect is statistically significant. Hence, H6 was supported by the analysis. The indirect effect of JE between PC and JP was 0.0137. The confidence interval did not include 0 (Boot LLCI = 0.0049, Boot ULCI = 0.0235), indicating that the mediating effect is statistically significant, meaning it is relative to H7. The indirect effect of PCF and JE between PC and JP was 0.0103. The confidence interval did not include 0 (Boot LLCI = 0.0038, Boot ULCI = 0.0172), indicating that the chain mediation effect is statistically significant, which buttressed H8 as depicted in Table 7.

**Table 7:** The mediating and chain mediating effect of problem focused coping and job engagement

Effect	Hypothesis	Model pathway	Indirect effect	BootSE	Boot LLCI		Boot ULCI	t- statistic	Decision
Indirect effect path 1	H6	PC→PCF→JP	0.0225	0.0078	0.0037	0.0268		2.884	Supported
Indirect effect path 2	H7	PC→JE→JP	0.0137	0.0047	0.0049	0.0235		2.914	Supported
Indirect effect path 3	H8	PC→PCF→JE→JP	0.0103	0.0034	0.0038	0.0172		3.029	supported

Note: Psychological capital (PC), Problem-Focus Coping (PFC), Job engagement (JE), and Job performance (JP)

## Discussion

The current quantitative study focuses on a survey-based analysis indicating the effects psychological capital a personal resource has on nurses’ problem focus coping to the extent of enhancing their performance in Cameroon’s public health facilities. The positive relationship between nurses’ psychological capital and their problem-focus coping is explained by the fact that nurses actively evaluate difficult circumstances and identify alternate solutions by relying on valuable energy-enhancing personal resources. This enables them to interpret difficult situations faced in the health institution constructively. This current study’s relational outcome is in line with studies done by H Wang, TK Ng and O-l Siu [8] and OL Siu, BCY Lo, TK Ng and H Wang [53] in which highlighted the importance of positive psychology in overcoming obstacles. This underscores the importance of exploring potential limiting factors that could clarify the situation, extending past research [8]. First, based on our empirical data, positive psychological capital boosts the opportunistic perception of difficulties, consistent with the COR theory [14] stipulating that; the high presence of positive personal resources leads to resource caravan which is rather beneficial for individuals since difficulties are perceived as opportunities.

Again, nurses can utilize their resources to motivate themselves to be more engaged. The findings of our study show that nurses’ psychological capital manifested in the form of optimism, resilience, self-efficacy, and hope fosters nurses’ job engagement. Nurses make use of their intrinsic resources, such as their resilience and optimism, to remain motivated at work [119]. These findings are in tandem with M Mansour [120], whose study revealed the importance of psychological capital on employees’ job engagement. This strong significant association is in agreement with the COR theory, highlighting the continuous necessity for individuals to acquire new resources with the help of their intrinsic capital. Therefore, the high presence of positivity within nurses creates a motivational spirit.

Consistent with previous studies, the manifestation of psychological capital has been empirically evaluated as a value-added to an individual’s work performance. This study’s findings correspond with many others: the positive effect psychological capital has on job performance [121, 122]. The current study extends the existing literature to a known body of employees (nurses) within a different cultural zone. The significant relationship between nurses’ psychological capital and job performance is a result of their self-efficacy, which drives their desire to achieve their work [123]. The increase in job performance is equally attributed to their rippling optimism.

Furthermore, our findings evidence that problem-focus coping intensifies job performance in our study. This finding easily slips into the existing literature discoveries [31, 53, 124], in which they found and discussed the positive effect problem-focus coping has on job performance. In the desire to attain difficult objectives and attain personal growth, nurses use coping mechanisms as a means to accumulate resources that help them attain organizational objectives. This occurs as a result of their attitudes and beliefs, which are more direct, hence providing more accurate predictions for behaviors connected to performance [125]. This current study lengthens the present literature by evaluating how nurses’ problem-focus coping can enhance their job performance.

In addition, the positive relationship between job engagement and job performance is generally considered by scholars as a traditional correlation, since it has been found several times that job performance is an antecedent of job engagement [84]. In the study of BP Owens, WE Baker, DM Sumpter and KS Cameron [126], the results emphasized how nurses make use of their dedication and vigor which they manifest as engagement to enhance their performance. In line with the current study revealing a positive association between nurses’ job engagement and job performance. This significant relationship is because; when deciding on how to assign their physical, cognitive, and emotional energies at work, engaged nurses simply throw their whole selves into their roles, which they understand to include any activity that could potentially contribute to their effectiveness.

Furthermore, problem-focus coping is a mediator of the effect of psychological capital on job performance. Our study demonstrates that there is support for the mediating role of problem-focus coping consistent with the works of E Rabenu, E Yaniv and D Elizur [6]. This extant study results supplements the existing research on psychological capital and its potential to influence employees’ coping capacities and enhance employee performance. Nurses believe they can handle the circumstances and they are able to cope. This indicates that nurses make use of their inner resources to better understand and face organizational challenges, this repeated mechanism predicts their long-run performance.

Apart from the above discussed, job engagement was found to act as a mediator on the effect psychological capital has on job performance. Our findings revealed a significant relationship among these constructs in agreement with the research of G Alessandri, C Consiglio, F Luthans and L Borgogni [90]. This is possible because nurses use their intrinsic resources (resilience and optimism) as a source of personal motivation which tends to engage them more at work and as a result improve their performance. The manifestation of positive psychology by nurses has the potential to maintain their mental well-being, and foster positive attitudes, consequently enhancing their work engagement and overall performance.

Lastly, this study recorded support for the hypothesized chain mediation role problem-focus coping and job engagement play in the association between psychological capital and job performance. Nurses’ cognitive and behavioral processing of challenges is directly related to their engagement. Therefore, nurses make use of their psychological resources for a better interpretation of difficulties. Nurses perceive difficult situations in their workplace differently - some view the hardship as a motivation-driving challenge, while others see it as a growth opportunity to expand their skills. There is also a minority who disregard the difficulty altogether. Yet despite these varying outlooks, grappling with such demanding circumstances commonly strengthens nurses’ dedication to their role and fuels their job performance by directing their energies towards constructive objectives. The ability to reframe adversity, whether by approaching it as a surmountable test, a chance to learn, or simply focusing elsewhere, leads nurses to reinvest their efforts in ways that ultimately enhance their productivity [95]. A strong intrinsic motivation is considered an armor in nurses, which assists them in overcoming difficulties and, in return, results in personal satisfaction, hence fostering their performance.

Despite the knowledge expansion, our investigation revealed some shortcomings that point to future research opportunities. First, the study focused on nurses in a specific public sector, which does not represent all healthcare settings. This implies that the findings in this study may not reflect happenings in the private health sector. The incorporation of additional personality traits like locus of control and openness to experience in the model would improve the comprehension of their moderating or mediating effects on problem-focus coping. Again, future studies should investigate these correlations in different sectors of activity, including hotels, restaurants, travel agencies, and airlines. According to RP Bagozzi and TF Heatherton [114], operationalized psychological capital and its four components using techniques such as the partial aggregation model shall give more accuracy to the result on the effects of psychological capital on coping behaviors, hence permitting scholars to make causal inferences. Segmenting engagement into its dimensions of vigor, dedication, and absorption [127] shall reveal detailed information on the importance and performance effect of problem-focus coping on job engagement and, subsequently, job performance. The study lacks an extensive qualitative feature. In-depth interviews could be used in subsequent research as a substitute approach to offer a greater understanding of the effects of psychological capital on problem-coping. Conducting this research in other parts of the world shall be an improvement to this model since coping behaviours can be influenced by family backgrounds, cultural ties etc. Finally, the study collected data at one point in time and longitudinal data could provide more insights into how psychological capital influences problem-focus coping over time.

### The theoretical implication of the research

This study makes significant theoretical contributions by applying the Conservation of Resources Theory and Job Demands-Resources Model to the healthcare context, specifically examining the relationships between psychological capital, problem-focused coping, job engagement, and job performance among nurses. These established theories provide a framework to understand how resources like psychological capital can positively impact coping, engagement, and outcomes [14, 21, 23]. Unlike previous studies that focused only on direct effects, this study delves into the underlying mechanisms through which psychological capital influences performance. It explores problem-focused coping and job engagement as potential mediators in the connection between psychological capital and job performance. By examining multiple mediators simultaneously rather than separately, it responds to the call for a more comprehensive approach [98]. The study offers a nuanced understanding of the sequential mediating pathways through which psychological capital exerts its influence.

Additionally, it extends organizational psychology and human resource management literature by conducting research in the healthcare context of Cameroon, a setting that has been understudied. The findings have the potential to enhance understanding of nurses’ experiences in the country and provide implications specific to the healthcare sector. Moreover, by incorporating problem-focused coping strategies as a mediator, the study bridges the gap between stress and coping literature and research on non-financial resources such as psychological capital. This advancement contributes to the theoretical understanding of the stress-buffering properties of personal resources and adaptive coping mechanisms. Overall, the study’s hypothesized model presents a comprehensive framework that elucidates how psychological resources and resilient behaviors interact as nurses navigate the demands of their jobs. It advances theoretical knowledge by exploring the antecedents and outcomes of psychological capital and the mechanisms that link individual strengths to job attitudes and work outcomes.

### The managerial implication of the research

This study has both employee and organizational-oriented practical implications worthy of elucidation. Focusing on the creation of psychological capital and addressing its impact on coping reactions of workers, this research has practical implications for medical facilities, looking to improve health personnel job performance. First, hospital managers have to implement green, sustainable human resource management to alleviate organizational challenges and uplift nurses’ psychological capital. It is, therefore, necessary for hospital administrators to implement a principle of fair-mindedness when it comes to organizational challenges; otherwise, it might be difficult for employees to reciprocate with positive behaviors and perform effectively. Managerial staff should focus on the negative symptoms or problems in individuals’ and organizations’ issues, such as conflict, stress, and depression, which characterize well-being and are more profitable to trigger positive psychological capital [128]. Effective implementation of a convenient remuneration system by civil administrators of Health and hospital management staff shall uplift the nurses’ psychological capital, causing them to ignore and screen some of the organizational difficulties but rather focus on workplace tasks, hence turning to be more productive [129]. Moreover, employees should make more use of their resources to face their work environment challenges, reciprocate with positivity to all work stresses and difficulties, and adopt favorable attitudes and behavior.

## Conclusion

This study investigated the relationships between psychological capital, problem-focused coping, job engagement, and job performance among nurses in Cameroon. Drawing upon the JD-R model and COR theory, the results supported most of the hypothesized relationships. Specifically, psychological capital was found to positively relate to problem-focus coping, job engagement, and job performance, demonstrating its role as an important personal resource for nurses.

Additionally, problem-focused coping and job engagement partially mediated the impact of psychological capital on job performance. This suggests that psychological capital may influence performance through strengthening nurses’ coping abilities and engagement in their work. The full chain mediation analyses also indicated that problem-focus coping and job engagement acted as sequential mediators in the association between psychological capital and job performance. These findings offer valuable insights for healthcare managers seeking to promote nurse well-being and performance. Nurturing positive psychological attributes like hope, self-efficacy, resilience and optimism can help equip nurses to better manage workplace demands through adaptive coping strategies and engagement in their roles. Maintaining high levels of personal resources may also help sustain nurse motivation and performance over time amid challenging conditions.

In conclusion, this study contributes fresh perspectives on the mechanisms linking individual and organizational resources to key healthcare workforce outcomes. The results highlight the importance of fostering personal resources like psychological capital that can help nurses adaptively deal with stress and remain motivated in their critical roles. These findings make theoretical contributions by demonstrating how psychological capital may act as a personal resource to help employees constructively navigate challenging workplace situations to remain dedicated and effective. The study also elucidates the mechanisms linking psychological traits to work behaviors. While novel, results should be considered with limitations, including the cross-sectional design and use of self-report measures. Further research across healthcare roles, organizations, and cultures using longitudinal approaches would prove beneficial.

## Data Availability

The datasets generated during and analyzed during the current study are available from the corresponding author on reasonable request.

## Abbreviations

PC: Psychological capital
PFC: Problem-focus coping
JE: Job engagement
JP: Job performance
